# Preoperative imatinib mesylate (IM) for huge gastrointestinal stromal tumors (GIST)

**DOI:** 10.1186/s12957-017-1143-2

**Published:** 2017-04-11

**Authors:** Sumin Tang, Yuan Yin, Chaoyong Shen, Jiaju Chen, Xiaonan Yin, Bo Zhang, Yuqin Yao, Jinliang Yang, Zhixin Chen

**Affiliations:** 1 Department of Gastrointestinal Surgery, West China Hospital, Sichuan University, Chengdu, 610041 Sichuan China; 2 Department of Research Center for Public Health and Preventive Medicine, West China School of Public Health/No. 4 West China Teaching Hospital, Sichuan University, Chengdu, 610041 Sichuan China; 3 Department of State Key Laboratory of Biotherapy/Collaborative Innovation Center for Biotherapy, West China Hospital, Sichuan University, Chengdu, 610041 Sichuan China

**Keywords:** Gastrointestinal stromal tumors, Preoperative imatinib mesylate, Progression-free survival, Overall survival

## Abstract

**Background:**

Preoperative imatinib mesylate (IM) treatment has not yet been standardized. Here, we aim to further explore such therapy on patients with gastrointestinal stromal tumors (GIST) retrospectively.

**Methods:**

Patients experiencing preoperative IM were identified from January 2009 to February 2015.

**Results:**

A total of 28 GIST patients were identified. The patients received preoperative IM treatment for a median length of 13.5 months, ranging from 5 to 37 months. PR and SD were observed in 24 (85.7%) and 4 (15.3%) patients, respectively. The tumor shrinkage occurred predominantly within 6 to 12 months, and slight tumor shrinkage could be observed after 12 months in certain patients. Nineteen patients (67.9%) received surgery, and R0 resection was acquired in 18 (94.7%) patients. The initial mean maximum diameter was 10.5 (5.2 to 19.0) cm and decreased to 5.9 (2.7 to 19.0) cm after preoperative treatment with a median length of 12 (ranging from 5 to 36) months (*P* < 0.001) in patients receiving operations. Three in 7 cases of rectum GIST underwent abdominoperineal resection, and four others adopted sphincter-sparing resection. Partial gastrectomy was performed in four patients.

**Conclusions:**

IM prior to surgery can effectively prevent tumor rupture and facilitate surgery with low surgical morbidity for GIST patients. Tumor shrinkage following IM occurred predominantly within 6 to 12 months, and slight tumor shrinkage could be observed after 12 months in certain patients. In selected patients, prolonged exposure to IM is seemingly advisable under close radiological surveillance.

## Background

Gastrointestinal stromal tumors (GIST) are the most common mesenchymal tumors of alimentary tract, accounting for approximately 1 to 3% of all malignant gastrointestinal neoplasms [1]. Surgery is the mainstay of localized GIST treatment for curative intention. The majority of GIST patients are candidates for complete resection of tumors at the first presentation. Nonetheless, a poor survival rate was acquired during follow-up before imatinib era, as demonstrated by a study of 1458 patients [2], let alone among cases with advanced or metastatic disease.

Imatinib mesylate (IM) is applied in GIST patients gradually based on the discovery that GIST is initiated by mutational proto-oncogene *KIT* or *PDGFRA* encoding receptor tyrosine kinase, which revolutionizes the management of the disease with its high efficacy, primary resistance observed in only approximately 10% patients [3]. Surgical resection combined with adjuvant IM has greatly improved the outcome of patients with intermediate or high relapse risk. Of note, when lesions are large or in tricky locations (e.g., low rectum, gastroesophageal junction, duodenum) or tend to rupture, immediate tumor removal is seemingly inadvisable because it could damage surrounding organs, entailing permanent lifestyle change or intraperitoneal tumor dissemination. In this case, IM may effectively downsize tumor to avoid these possible undesirable consequences.

However, the duration of preoperative IM is still unclear. The NCCN guideline recommends a maximal response time 6 to 12 months after IM onset. Multidisciplinary team members should collaborate to determine whether operation is needed when early response is acquired in some cases without giving definite time frame in unresectable or locally advanced primary GIST [4]. Both 6 months of preoperative IM [5, 6] and waiting for stagnation of tumor shrinkage detected by consecutive radiographic imaging [7] in the recurrent/metastatic setting are also proposed. On the other hand, survival benefit from an operation following IM cannot be determined. Considering the above uncertainties, we aim to further investigate the role of IM prior to surgery in the current study retrospectively to provide reference for its rational use in clinical practice.

## Methods

### Patient selection

Patients experiencing preoperative IM were identified from West China Hospital, Sichuan University, over a continuous period, from January 2009 to February 2015. The following is the inclusion criteria: (1) patients were pathologically confirmed as primary or recurrent/metastatic GIST stained positive for CD117 and/or Dog-1, whose specimens were obtained by fine needle biopsy or surgery or endoscope; (2) GIST were diagnosed to be unresectable, or more mutilating surgery was needed due to locally advanced or metastatic disease or rupture tendency, assessed by a multidisciplinary team of experienced radiologists, oncologists, and surgeons; and (3) patients with recurrent/metastatic disease who had no history of taking IM. The following is the exclusion criteria: (1) GIST showed primary resistance to IM in gene analysis or radiographic imaging, and (2) patients had poor performance status due to other serious diseases. The Institutional Review Board and Ethics Committee of the West China Hospital of Sichuan University deemed that an ethical review was not needed for this retrospective research.

### Definitions

The response of target lesions to IM treatment was defined in our study cohort according to the revised RECIST guideline (version 1.1) [8]. Plateau was defined as no evidence of change in size detected by two consecutive radiographic imaging. Margin status: R0 resection was defined as a complete removal of tumor tissue with microscopically negative margin, R1 resection was defined as a removal of tumor tissue with microscopically positive margin or tumor intraoperative rupture, and R2 resection was defined as an incomplete removal of tumor tissues with macroscopically positive margin. Time: follow-up time was calculated from the date of initiation of IM treatment to the date of the last follow-up or death of patient, progression-free survival (PFS) time was calculated from the date of initiation of IM treatment to the date of occurrence of tumor progression or the last follow-up or death of patient, and overall survival (OS) time was calculated from the date of initiation of IM treatment to the date of the last follow-up or death of patient.

### IM treatment and operation timing

IM was administrated as a first-line drug treatment at a dose of 400 mg once daily. Surgical excision of tumors was performed as tumor progressed or tumor downsizing plateaued, or multidisciplinary team deemed improved surgery (radical and/or organ-sparing resection) possible.

### Follow-up and data collection

Relevant demographics, tumor characteristics, pathological reports, and treatment protocols were collected. Consecutive baseline computed tomography (CT) or magnetic resonance imaging (MRI), abdominal ultrasonography, and full hematological test were performed on every patient after the initiation of IM treatment during the follow-up period. If liver parenchyma was invaded by GIST, liver function assays were also included. Imaging examination intervals depended on the attending physician, or the patient and results would be recorded in detail. The last follow-up was in October 2015 or the date when patient died of GIST progression or other causes.

### Statistical analysis

Count data were shown as percentages while measurement data as mean or median with the range from the minimum to the maximum. The paired sample or independent sample *t* test was used to analyze difference of tumor size as appropriate. Difference of follow-up periods between operation and non-operation groups was identified by Mann-Whitney *U* test. PFS and OS were analyzed by the statistical method of Kaplan and Meier, and univariate analysis was performed using the log-rank test. A two-sided *P* value less than 0.05 was considered statistically significant. Death resulting from other causes rather than GIST was counted as censoring. The statistical software of SPSS 17.0 (SPSS, Chicago, IL, USA) was used to analyze all raw data.

## Results

### Demographics and clinicopathologic data

A total of 28 patients were included with male to female ratio being 21:7. Maximum tumor diameter was calculated with CT and MRI, and all exceeded 5 cm with >8.3 cm accounting for 75% prior to IM treatment. The majority of tumors were primarily located in the small intestine (32.1%), followed by the stomach (28.6%), the rectum (21.4%), other (10.7%), and the colon (7.1%). There were eight patients carrying multiple GIST lesions, and four were accompanied by synchronous liver metastases among them. Risk stratification was done according to the Modified NIH criteria [9]. All patients were classified as high-risk group for disease relapse. Gene mutation analysis was conducted in only 13 patients, and the distribution was 9 in exon 11, 3 in exon 9, and 1 in WT. Other relevant characteristics of patients and tumors are listed in Tables 1 and 2.

**Table 1:** Demographics and clinicopathologic characteristics of all patients

Characteristics	Patients *n* (%)
Median age, years (range)	52 (28–76)
Sex	
Male	21 (75.0)
Female	7 (25.0)
Mean initial maximum diameter (range), cm	10.7 (5.2–19.0)
Primary location	
Stomach	8 (28.6)
Small intestine	9 (32.1)
Colon	2 (7.1)
Rectum	6 (21.4)
Other	3 (10.7)
CD117 positivity	26 (92.9)
CD34 positivity	21 (75.0)
Median follow-up (range), months	21 (8–75)

**Table 2:** Main characteristics of operated patients

Patient no.	Gender	Age (years)	P/R	Number of lesions (single/multiple)	Lesion location	Preoperative TKIs duration (months)	Surgical procedure	Margin status
1	M	57	P	Single	Stomach	18	Partial gastrectomy combined with spleen resection	R0
2	M	44	P	Single	Small bowel	19	Partial small bowel resection	R0
3	M	39	R	Multiple	Small bowel and omentum	9	Tumor resection combined with partial small bowel resection	R0
4	M	41	R	Multiple	Small bowel and liver	6	Partial small bowel resection combined with liver RFA	R2
5	M	63	R	Multiple	Omentum and mesentery of small bowel	18	Tumor resection	R0
6	M	46	P	Single	Rectum	6	Abdominoperineal resection	R0
7	M	35	P	Single	Colon	13	Partial colon resection	R0
8	F	52	P	Single	Rectum	5	Abdominoperineal resection	R0
9	M	37	R	Single	Rectum	12	Abdominoperineal resection	R0
10	M	28	R	Multiple	Colon and small bowel	36	Partial colon and small bowel resection	R0
11	F	60	P	Single	Small bowel	12	Partial small bowel resection	R0
12	F	61	P	Single	Rectum	12	Sphincter-sparing resection by laparoscope	R0
13	M	51	P	Single	Rectum	24	Sphincter-sparing resection	R0
14	M	57	P	Single	Small bowel	8	Partial small bowel resection	R0
15	M	44	R	Single	Rectum	36	Sphincter-sparing resection	R0
16	F	59	P	Single	Rectum	10	Sphincter-sparing resection	R0
17	F	55	P	Single	Stomach	13	Partial gastrectomy	R0
18	F	53	P	Single	Stomach	9	Partial gastrectomy	R0
19	M	43	P	Single	Stomach	12	Partial gastrectomy	R0

*M* male, *F* female, *P/R* primary/recurrent or metastatic, *RFA* radiofrequency ablation

### Response to IM treatment

**Fig. 1:**
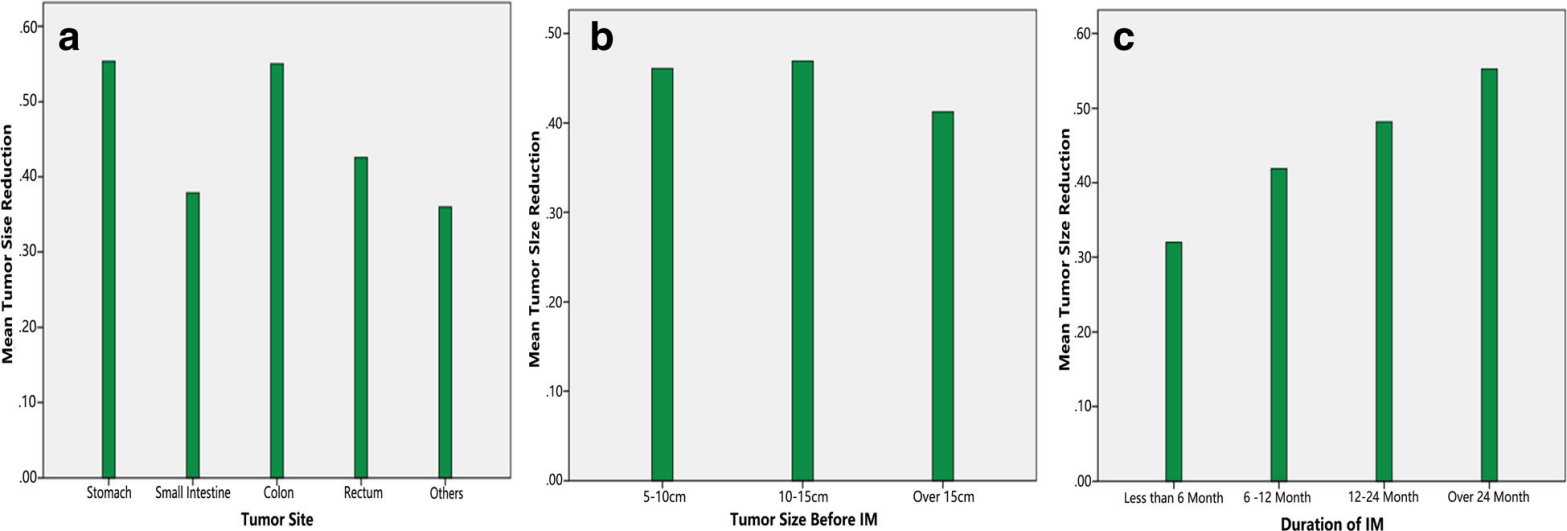
Mean tumor size reduction (%) of different sites of tumor (**a**), initial tumor maximum diameters (**b**), and durations of IM (**c**)

**Fig. 2:**
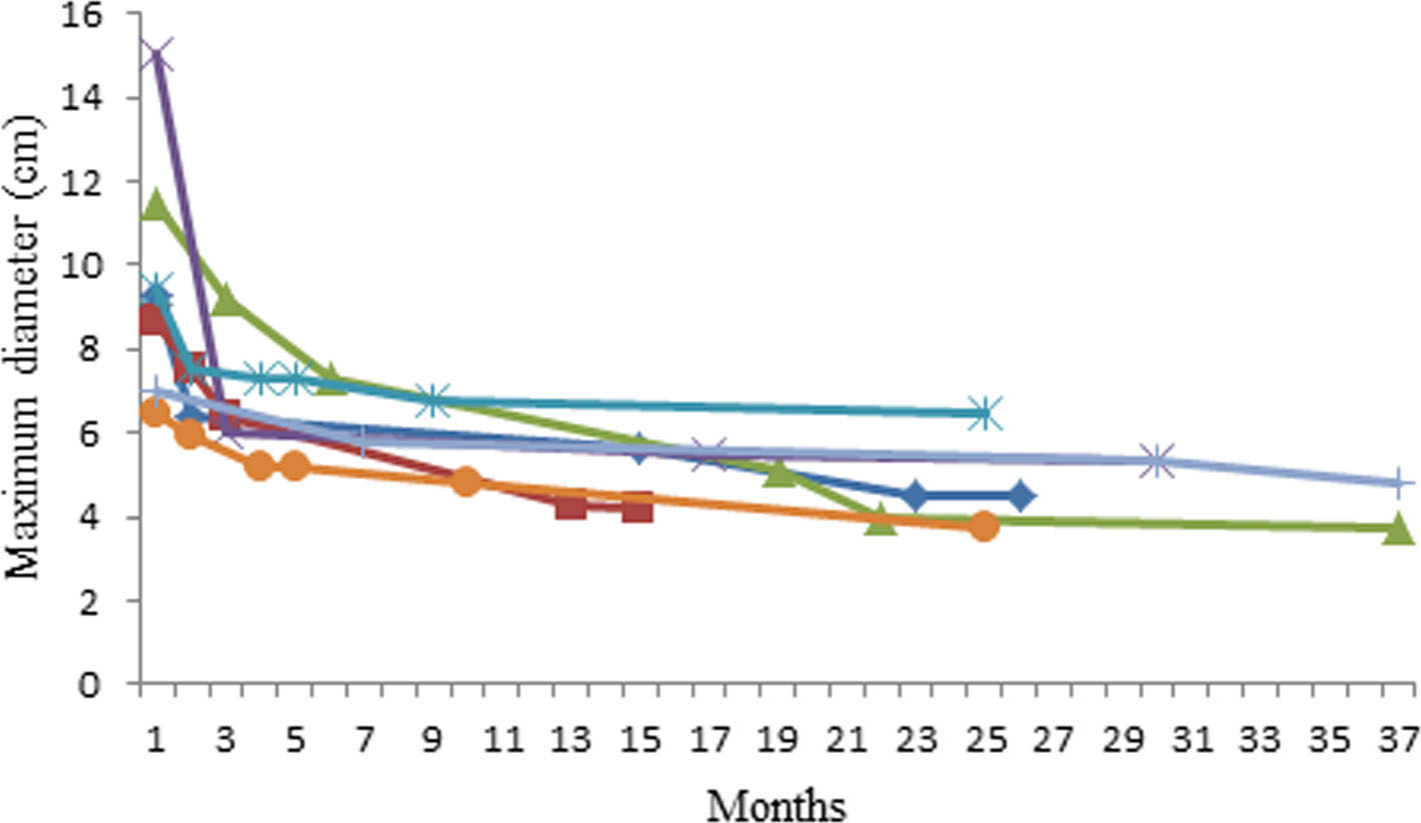
Successive changes of tumor maximum diameters in seven patients receiving more than a 12-month IM

All patients were administrated with first-line drug IM at a dose of 400 mg once daily. The patients received preoperative IM treatment for a median length of 13.5 months, ranging from 5 to 37 months. PR and SD were observed in 24 (85.7%) and 4 (15.3%) patients, respectively. The mean maximum diameter of tumor decreased from 10.7 (5.2 to 19.0) cm to 5.9 (2.7 to 19.0) cm (*P* < 0.001). No primary drug resistance was documented. No severe drug-related side effect took place in this series. No obvious association between the mean tumor size reduction and location of tumor (Fig. 1a) or the initial tumor maximum diameter (Fig. 1b) was observed. It seemed that with longer IM duration, the tumor tended to shrink greatly, but still the tumor shrinkage occurred predominantly within 6 to 12 months, slight tumor shrinkage could be observed after 12 months in certain patients (Figs. 1c and 2). In most cases, the tumor stopped shrinking after 12 month and stayed stable for as long as 2 years (Fig. 1c).

Nineteen patients (67.9%) finally received surgical resection of tumor following preoperative IM with a median length of 12 (5 to 36) months. PR and SD were demonstrated in 16 and 3 patients, respectively. The initial mean maximum diameter was 10.5 (5.2 to 19.0) cm and decreased to 5.9 (2.7 to 19.0) cm after preoperative treatment (*P* < 0.001). All of the 19 patients were given adjuvant IM due to high recurrence risk after surgery.

The remaining 9 patients refused operation and chose only IM treatment with a median length of 24 (8 to 37) months. The mean maximal diameter of tumor reduced from 11.0 (9.0 to 15.6) cm to 5.9 (3.7 to 8.0) cm (*P* < 0.001).

### Surgery information

Eventually, 13 (81.3%) primary and 6 (50%) recurrent/metastatic patients experienced surgical procedure. R0 resection was acquired in 18 (94.7%) patients, and minor lesions in the liver could not be removed completely in patient no. 4. No tumor rupture and blood transfusion at operation happened. Three in 7 cases of rectum GIST underwent abdominoperineal resection because the anus was too adjacent to the tumor to be preserved, and sphincter-sparing resection of tumors was adopted in 4 (57.1%) cases with excision margins pathologically negative, which was impossible initially. Partial gastrectomy was realized in 4 (100%) patients with huge mass in the stomach which could not be resected or potentially resectable with total or subtotal gastrectomy, before taking IM, judged by radiological findings or exploratory laparotomy. The remaining eight patients with tumors in other parts of the body were also performed organ-preserving or less-mutilating surgery as anticipated. Pulmonary infection and anastomotic fistula occurred separately in two cases, which were managed well through prolonged hospitalization care and fistula repair. Detailed information is recorded in Tables 2 and 3.

**Table 3:** Correlation between status of surgical resection and tumor progression/death

Variables	Progression *n* (%)	Death *n* (%)
Overall (*n* = 28)	4 (14.3)	2 (7.1)
Surgery (*n* = 19)	2 (10.5)	0 (0)
No surgery (n = 9)	2 (22.2)	2 (22.2)
Primary (*n* = 16)	1 (6.3)	0 (0)
Surgery (*n* = 13)	1 (7.7)	0 (0)
No Surgery (*n* = 3)	0 (0)	0 (0)
Recurrent/metastatic (*n* = 12)	3 (25.0)	2 (16.7)
Surgery (*n* = 6)	1 (16.7)	0 (0)
No Surgery (*n* = 6)	2 (33.3)	2 (33.3)

### Prognosis

**Fig. 3:**
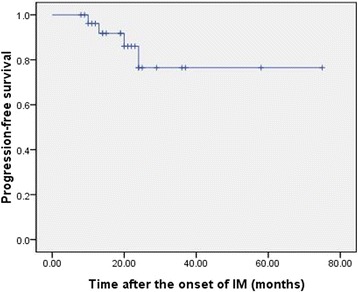
Kaplan-Meier curve of PFS for 28 patients

No patients were lost in the follow-up period, and the median follow-up was 21 (8 to 75) months from the onset of IM treatment. The rate of 2-year PFS was 76.5% (Fig. 3). No death took place among patients taking the surgery during the follow-up, and tumor progressed in two cases at the time of 24 and 13 months (15 and 5 months after operation), respectively, for whom further operation was adopted in combination with postoperative sunitinib. Among patients refusing surgery, one died at 24 months and another at 15 months because of tumor progression, the two patients had stopped taking IM and any other drugs for 4 and 5 months before disease progression. The differences of follow-up periods and tumor maximum diameters at baseline between operation and non-operation groups were not statistically significant (*P* = 0.980, 0.741 respectively), PFS and OS as well (*P* = 0.513, 0.077 respectively). Median PFS and OS had not been reached at the last follow-up. Detailed information is recorded in Table 3.

## Discussion

Preoperative IM treatment is playing an indispensible role in locally advanced or recurrent/metastatic GIST by contributing to complete tumor resection with the least cost. Until now, multiple studies have delineated benefits from the treatment, for example, low surgical morbidity and mortality and function-preserving [10–13]. It is further confirmed by the current study enrolling 28 patients with PR and SD observed in 24 (85.7%) and 4 (15.3%) cases, respectively, in which R0 resection was acquired in 18 patients by less extensive surgical procedure without occurrence of tumor rupture or hemorrhage owing to tumor shrinkage and loss of vascularity. Even three patients harboring mutations in exon 9 and 1 in WT showed good response to IM of 400 mg once daily.

The optimal duration of preoperative IM or other kinase inhibitors remains controversial. The major concern is missing a surgery window if long-term IM treatment allows tumor to progress, while short-term IM treatment cannot achieve optimal efficacy. During the past years, reported durations of IM prior to surgery tended to be inconsistent, varying between a few days and several months. The RTOG 0132 trial probed into preoperative IM effectiveness after a period of 8 to 12 weeks, followed by 2-year adjuvant IM for advanced primary (group A) and recurrent/metastatic patients (group B), with 2-year PFS of 83 and 77% for a separate group, and the results of the trial were updated in 2012 [14, 15]. The median duration was 7.3 months among 9 patients receiving surgical resection of lesions in another report, which involved 25 locally advanced patients identified from the prospective BFR14 trial [16].

Our patients in this study experienced a longer IM duration before operation (a median duration of 13.5 months, ranging from 5 to 37 months) compared to earlier reports. Figure 2 demonstrated successive changes of maximum tumor diameters in seven patients receiving more than a 12-month IM. A rapid tumor downsizing was observed within 6 months of preoperative treatment, and shrinkage can still be observed after 12 months in some cases but slower. In most cases, the tumor stopped shrinking after 12 months and stayed stable for as long as 2 years, which was displayed in Fig. 1. Eventually, R0 resection was done in more than 90% patients and in all primary patients. The R0 resection rate is higher than 83 and 88.2% presented separately by the EORTC STBSG experience with a median time of 40 weeks for preoperative IM [17] and the phase II APOLLON trial with a median time of 27 weeks [18]. We could not conclude that prolonged preoperative IM facilitate R0 resection, but at least suggest that prolonged preoperative IM do not always lead to disease progression, which makes delayed surgery possible for patients of poor performance status who did not tolerate operation. In a particular case, even sunitinib can be administrated as a replacement of IM in the preoperative setting when tumors develop resistance to IM [19].

Preoperative IM could promote safety of GIST surgical resection by facilitating tumor shrinkage and loss of vascularity [20, 21]. As revealed by the current study, although GIST is a kind of hypervascular tumor with a tendency to be fragile, no tumor rupture or blood transfusion occurred at operation among 19 operated patients. Organ-preserving or less-mutilating surgery was also realized. Sphincter-sparing resection of tumors was adopted in 4 of 7 (57.1%) cases with excision margins pathologically negative, and 4 (100%) gastric GIST patients experienced partial gastrectomy, which was impossible before taking IM, judged by radiological findings or exploratory laparotomy. It has been reported by Demetri et al. [22] that IM plasma trough levels (*C*_min_), which is affected by transit time and PH in the gastrointestinal tract, is related to the prognosis of advanced GIST. IM *C*_min_ of patients experiencing total or subtotal gastrectomy was lower than that of patients experiencing partial or no surgery [23]. Therefore, preoperative IM and less extensive operation in patients with gastric GIST may have an additional benefit on adjuvant IM treatment after surgery since a higher IM *C*_min_ may be achieved. Of note, laparoscopic surgery by experienced surgeons should be available after preoperative IM treatment.

In this series, a 2-year PFS rate of 76.5% was demonstrated, which was similar to previous reports [14]. Nine patients refused operation for financial reasons or other, even a R0 resection seems possible after preoperative IM. In 19 operated patients, 6 (31.6%) were diagnosed as recurrent or metastatic GIST. There were no statistically significant differences between patients with and without surgical resection regarding PFS (*P* = 0.513) and OS (*P* = 0.077) in our study at the time of last follow-up. However, the previous research revealed that surgery following IM could improve both PFS and OS (*P* = 0.0318, 0.0217 respectively) in locally advanced non-metastatic GIST patients [16]. An interesting question is whether recurrent or metastatic patients with a good response to imatinib will receive any further benefit from surgical resection. Given our study’s small and non-randomized sample, definite answer cannot be made. Survival benefits of preoperative IM may also not be identified. But improvement of survival could be anticipated since IM prior to surgery can prevent tumor rupture, facilitate surgery with low surgical morbidity and may alleviate influence on IM *C*_min_. Of course, randomized controlled trial of large sample accompanied by long-term follow-up should still be warranted to further investigate preoperative IM.

## Conclusions

In summary, tumor resection was R0 in more than 90% patients after IM for a median length of 12 months and low surgical morbidity was observed. Accordingly, we suggest preoperative IM when GIST is large in size or undesirable in location. In selected patients, prolonged exposure to IM is seemingly advisable under close imaging surveillance.

## Abbreviations

GIST: Gastrointestinal stromal tumors
IM: Imatinib mesylate
OS: Overall survival
PFS: Progression-free survival
